# Millimeter-Scale Patterns of Phylogenetic and Trait Diversity in a Salt Marsh Microbial Mat

**DOI:** 10.3389/fmicb.2012.00293

**Published:** 2012-08-10

**Authors:** David W. Armitage, Kimberley L. Gallagher, Nicholas D. Youngblut, Daniel H. Buckley, Stephen H. Zinder

**Affiliations:** ^1^Department of Integrative Biology, University of California BerkeleyBerkeley, CA, USA; ^2^Department of Marine Sciences, University of ConnecticutGroton, CT, USA; ^3^Department of Microbiology, University of Illinois at Urbana-ChampaignUrbana, IL, USA; ^4^Department of Crop and Soil Sciences, Cornell UniversityIthaca, NY, USA; ^5^Department of Microbiology, Cornell UniversityIthaca, NY, USA

**Keywords:** microbial mat, community assembly, biodiversity, phylogenetics, null models, metagenomics, salt marsh

## Abstract

Intertidal microbial mats are comprised of distinctly colored millimeter-thick layers whose communities organize in response to environmental gradients such as light availability, oxygen/sulfur concentrations, and redox potential. Here, slight changes in depth correspond to sharp niche boundaries. We explore the patterns of biodiversity along this depth gradient as it relates to functional groups of bacteria, as well as trait-encoding genes. We used molecular techniques to determine how the mat’s layers differed from one another with respect to taxonomic, phylogenetic, and trait diversity, and used these metrics to assess potential drivers of community assembly. We used a range of null models to compute the degree of phylogenetic and functional dispersion for each layer. The SSU-rRNA reads were dominated by Cyanobacteria and Chromatiales, but contained a high taxonomic diversity. The composition of each mat core was significantly different for developmental stage, year, and layer. Phylogenetic richness and evenness positively covaried with depth, and trait richness tended to decrease with depth. We found evidence for significant phylogenetic clustering for all bacteria below the surface layer, supporting the role of habitat filtering in the assembly of mat layers. However, this signal disappeared when the phylogenetic dispersion of particular functional groups, such as oxygenic phototrophs, was measured. Overall, trait diversity measured by orthologous genes was also lower than would be expected by chance, except for genes related to photosynthesis in the topmost layer. Additionally, we show how the choice of taxa pools, null models, spatial scale, and phylogenies can impact our ability to test hypotheses pertaining to community assembly. Our results demonstrate that given the appropriate physiochemical conditions, strong phylogenetic, and trait variation, as well as habitat filtering, can occur at the millimeter-scale.

## Introduction

The mounting evidence that biodiversity *per se* positively affects the emergent functions of an ecosystem justifies further studies of the mechanisms by which taxa coexist (Loreau et al., 2001; Hooper et al., 2005). Much of this theory is built on data from eukaryotes, due mainly to our inability to survey bacterial, archaeal, and viral (henceforth microbial) assemblages in their natural environments (but see Bell et al., 2005). Beyond driving a number of critical biogeochemical functions, microbes encompass a tremendous pool of undescribed biodiversity on earth (Curtis and Sloan, 2004; Quince et al., 2008). Censuses of microbial diversity commonly encounter staggering levels of genetic and taxonomic information, and often lead to the discovery of novel biological functions (Cowan et al., 2005). In particular, the metagenomic and targeted-gene amplicon approaches to microbial ecology can be combined to visualize and statistically compare multiple dimensions of biodiversity within and between environmental samples (Tyson et al., 2004; Sogin et al., 2006).

Biodiversity within a microbial community can be defined in three fundamental ways. Taxonomic diversity, or the number of arbitrarily similar units and their abundance distributions, is the traditional metric by which communities are defined and compared. Trait diversity, or the breadth of phenotypic, rather than genotypic, differences among individuals also has a rich history of use in ecology. Certain traits are used to signify the role an organism plays in the context of biotic and abiotic interactions. These traits are termed “functional” in the sense that they influence the properties of the greater ecosystem. Because traits are a product of evolutionary dynamics within a population, a phylogenetic perspective on diversity is a useful bridge between taxonomic and trait diversity (Faith, 1992).

It is now recognized that communities are dynamic, arbitrarily bounded assemblages whose members are products of both contingent, historical processes, and semi-deterministic assembly rules (Ricklefs, 2006). Historical processes that structure communities include the constraints on diversification imposed by biogeography (e.g., dispersal limitation) and evolution (e.g., adaptive radiation, Red Queen dynamics; MacArthur and Wilson, 1967; Vermeij, 1987; Losos et al., 1998; Gillespie, 2004). In contrast, assembly rules are generally defined as contemporary mechanisms which either permit or prohibit an individual or taxon from occupying a particular local habitat (Diamond, 1975; Weiher and Keddy, 1999). While phylogenetic approaches have been a cornerstone in evolutionary biogeography for some time, ecologists have only recently adopted a phylogenetic perspective to tease apart community assembly mechanisms (Webb et al., 2002; Cavender-Bares et al., 2004). Most commonly, the phylogenetic relatedness (or trait similarity) of taxa within a particular habitat is compared to the averaged relatedness of randomized communities whose taxa are sampled from a pool of potential colonizers (called a *regional pool*). If the observed phylogenetic relatedness of a community is significantly lower or higher than the mean (or median) of the randomized communities, then the community is said to be either phylogenetically clustered or overdispersed, respectively (Webb, 2000). If the assumption of phylogenetic niche conservatism is justified, then the differences between two organisms’ traits should positively covary with phylogenetic distance (Peterson et al., 1999). Thus, if habitat filtering is a dominant community assembly mechanism, we expect to find phylogenetic and trait clustering in that habitat due to trait-driven niche conservatism. Alternatively, in a scenario involving character displacement, competition between sister taxa will result in divergent selection on their traits (and hence their realized niches). In this case, habitat filtering results in independence between the phylogenetic diversity and trait diversity of a community, and manifests as functional clustering with no pattern to the phylogenetic structure of the community.

The assumption of phylogenetic niche conservatism appears robust at very broad taxonomic levels (e.g., among all Angiosperms), but can break down within smaller clades (e.g., oak species; Cavender-Bares et al., 2006). In microbial communities, horizontal gene transfer (HGT) among distantly related taxa weakens such an assumption at all taxonomic levels. Conversely, certain mono- and polyphyletic clades do possess a suite of traits which make certain habitats much more favorable. For example, the Cyanobacteria require both light and oxygen to carry out photosynthesis and respiration, and thus should generally be found in oxic, photic habitats. Likewise, the microaerophilic and anaerobic non-oxygenic phototrophs require light of particular wavelengths, as well as reduced inorganic sulfur and hydrogen for photosynthesis. Many obligate anaerobes (e.g., order Clostridiales) can similarly be found in habitats satisfying certain abiotic conditions.

Metagenomic shotgun and targeted-gene amplicon sequencing are two complementary culture-independent approaches to assessing microbial biodiversity. The former offers a relatively unbiased view of the suite of genomic information in an environmental sample, provided adequate sequencing depth and assembly steps. Assembled sequence fragments can then be compared against reference databases to predict their structures and potential functions. The downsides to this approach include erroneous predictions of gene function and limitations assessing overall diversity due to inadequate sequencing depth. To overcome the second caveat, phylogenetically informative genes (e.g., SSU-rRNA) can be amplified by PCR and the resulting amplicon pool sequenced alongside or independent of a shotgun metagenome. Depending on the quality and length of shotgun contigs and amplicon sequences, both metagenomic and amplicon approaches can yield information on the taxonomic, phylogenetic, and functional aspects of microbial biodiversity (Burke et al., 2011). Consequently, these data can also be used to inform our understanding of the processes structuring microbial communities. For instance, many studies have found evidence for phylogenetic and functional clustering in microbial assemblages in marine (Barberán and Casamayor, 2010; Kembel et al., 2011; Bryant et al., 2012; Pontarp et al., 2012), freshwater (Horner-Devine and Bohannan, 2006; Newton et al., 2007; Amaral-Zettler et al., 2010; Barberán and Casamayor, 2010), and terrestrial habitats (Horner-Devine and Bohannan, 2006; Bryant et al., 2008; Wang et al., 2012). Although these results are often taken as evidence for habitat filtering, many are based on comparisons of samples collected at distances which are often orders-of-magnitude greater than the scale at which cells are known to interact (Long and Azam, 2001; Dechesne et al., 2006). Therefore, quantification of niche-based community assembly may be confounded by historical biogeographic and evolutionary processes, such as adaptive radiation, genetic drift, and serial founder effects (Ricklefs, 2006; Vamosi et al., 2009; Fine and Kembel, 2011). By measuring the phylogenetic and functional properties of adjacent habitats at a scale permissive of genetic admixture (so-called microhabitats), such results can be more reliably attributed to trait-driven differences in habitat specialization (Webb et al., 2008).

Microbial mats are one of the more conspicuous and well-studied microbial communities (Stal and Caumette, 1994; Seckbach and Oren, 2010). These mats typically form in habitats too extreme to support plant growth, such as hypersaline soils, geothermal springs, and tidal flats. Their laminated appearance is due to vertical segregation of particular guilds of bacteria and diatoms, which assemble in response to millimeter-scale gradients in both light intensity and redox potential (Jørgensen et al., 1979; Revsbech et al., 1983; van Gemerden, 1993). In temperate environments, the top layer is often dominated by oxygenic cyanobacteria and eukaryotic algae and takes on a green hue due to its chlorophyll *a* content. During daylight hours, the oxygen concentration of this layer is equal to or higher than atmospheric levels and decreases with depth to trace levels at 5 mm. Thus, this layer also supports a rich community of aerobic heterotrophs. The production of extracellular polysaccharides (EPS) in the upper layers, however, probably limits the efficacy of larger eukaryotic grazers in capturing prey (Awramik, 1984). Light becomes more diffuse past 3 mm depth, but can still drive non-oxygenic photosynthesis in groups such as purple sulfur bacteria and green sulfur bacteria, provided the appropriate reducing agents are available (Jørgensen and Des Marais, 1986; Pierson et al., 1990). At depths greater than 10 mm, light is absent at wavelengths <1 μm and photosynthesis does not occur. Here, the microbial community primarily consists of anaerobic sulfate-reducers, although this form of respiration also occurs in the mats’ photic zones (Pierson et al., 1987; Risatti et al., 1994).

Despite the historical significance of microbial mats, such as their role in the ecology of early Earth (Des Marais, 2003), there have been few molecular surveys of such communities, and even fewer focusing on temperate salt marsh habitats (Ley et al., 2006; Buckley et al., 2008; Kunin et al., 2008; Bolhuis and Stal, 2011; Burow et al., 2012). Our aim was twofold: (1) to present the results of a shotgun metagenomic and targeted-gene amplicon survey of a particularly well-studied salt marsh microbial mat and (2) determine if patterns in the taxonomic, phylogenetic, and functional diversities of the microbial mat show evidence for non-random community assembly. The extreme biotic stratification and abiotic gradients evident in microbial mats led us to predict systematic differences in microscale biodiversity. For instance, because light, oxygen, and sulfur gradients in the mat favor particular metabolic strategies, and since many of these metabolic (particularly photosynthetic) strategies are phylogenetically conserved, taxa present within each layer should be more related to one another than expected by chance, or phylogenetically clustered, when measured over the entire bacterial domain. Under the assumption of phylogenetic niche conservatism, if trait-based habitat filtering is a dominant mechanism of community assembly, functional traits should also be clustered. Alternatively, if HGT is not a product of phylogenetic distance, a widespread prevalence of non-homologous recombination should decouple phylogenetic and trait diversity patterns, and manifest as clustered traits with random phylogenetic dispersion.

## Materials and Methods

### Study site

**Figure 1:**
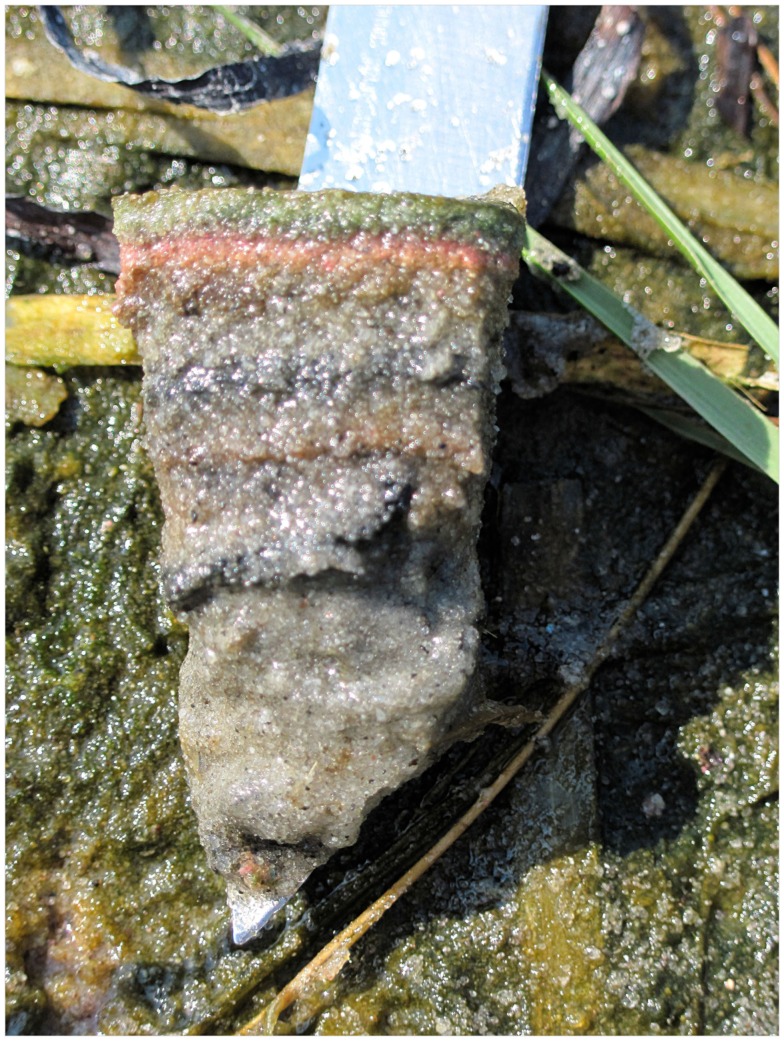
**Greater Sippewissett salt marsh microbial mat showing typical lamination (photo credit: NDY)**.

The Great Sippewissett salt marsh is located west of Falmouth, MA, on Buzzard’s Bay (N41°35′13.34″, W70°38′29.10″). The habitat is typical of New England salt marshes, with braided tidal creeks running around dense stands of *Spartina*. Microbial mats form in sandy intertidal sediments which lack colonization by plants, and are identifiable by the leathery, green/gold-colored top layer (Nicholson et al., 1987; Pierson et al., 1987; Figure 1).

We collected samples from the Great Sippewissett salt marsh on two occasions: June 23, 2010 and July 6, 2011. In 2010, two cores measuring 2 cm (diameter) × 5 cm were collected from two mat habitats. One of these cores was chosen as an example of an early successional mat. The early successional mat consisted of a wide light-green band of cyanobacteria descending from the surface to approximately 1 cm depth in unconsolidated sandy sediment. The early successional mat lacked a leathery surface layer, conspicuous bands attributable to anoxygenic phototrophs, and the cohesiveness which are all characteristic of climax mat communities in this system (Nicholson et al., 1987). The early successional mat was likely initiated at the end of winter and was developing in close proximity (1–3 m) to an area containing mature mats. Our second core from 2010 was taken from this climax mat system which consisted of a thick leathery surface layer and sharply defined dark green, pink, and brown cohesive layers (as described in Nicholson et al., 1987). Our assumption that these mat sections were of different ages is based on (1) their qualitative differences (slight green banding vs. multicolored layering, loose vs. stabilized sediment) and (2) their close proximity (similar biotic and abiotic characteristics). These definitions are consistent with descriptions of coastal mat development elsewhere (Stal et al., 1985; Mir et al., 1991; Stal and Caumette, 1994). Henceforth, we refer to the two cores from 2010 as “young mat” (YM-10) and “old mat,” (OM-10). We collected an additional core from Great Sippewissett salt marsh in 2011. This core was representative of a climax microbial mat community and is referred to as OM-11. Intact mat sections (20 cm^2^) were returned to the lab for processing. Cores were taken from the center of mat sections, the cores sectioned, and DNA extracted within 2–3 h of collection. The developed mats were sectioned with a sterile razor blade at the boundaries of their colored layers, and the young mat at corresponding depth.

Oxygen concentration and pH measurements were performed in 2011 using OX-50 and a pH-probes attached to a Microsensor Monometer (Unisense, Denmark). *In situ* depth profiling was conducted with a Unisense micromanipulator MM33. We also used abiotic data from previously published studies of the Sippewissett microbial mat including chlorophyll *a*, bacteriochlorophyll *a*, and sulfide (Pierson et al., 1987; Buckley et al., 2008). While these data are not perfectly matched to our samples, the descriptions and locations of the mats used in each study closely resemble our own.

### DNA extraction and sequencing

DNA was extracted from each sectioned layer using the MoBio PowerSoil kit according to the manufacturer’s instructions. This extraction was prepared for sequencing at the Bay Paul Center (Marine Biological Laboratory, Woods Hole, MA, USA) and the procedure is detailed elsewhere (Huber et al., 2007 Supplementary Materials). Briefly, this involved PCR amplification of the SSU-rRNA v4 through v6 hypervariable regions using degenerate primer sets 518F (5′-CCAGCAGCYGCGGTAAN-3′) and 1064R (consensus: 5′-CGACRRCCATGCANCACCT-3′) for the 2010 cores and primers 515F (5′-GTGYCAGCMGCCGCGGTAA-3′) and 907R (5′-CCGYCAATTCMTTTRAGTTT-3′) for the 2011 cores (Morrison and Sogin, Unpublished). The forward primers were synthesized to include (from 5′ to 3′) Roche A-adapters, a unique 8-base barcode specific to each sample, a 2-base linker sequence, and the forward primer. Reverse primers were synthesized with the Roche B-adapter linked to the primer via 2-base linker. Template concentration among all extractions was normalized to 15 ng/μl. PCR templates were amplified using 2× Phusion HF polymerase with 8% DMSO for 32–36 cycles, the first 10 cycles using a touchdown annealing temperature from 68 to 58°C followed by 12 cycles of three-step and 10–14 cycles of two step PCR. We used the PicoGreen assay to quantify PCR product and concentrate it to 100 μl. It was then gel-purified using the Montage Kit (Millipore) and shipped to Penn State Genomics Core Facility and sequenced on the Roche GS-FLX Titanium platform.

In addition to the SSU-rRNA amplicon libraries, we performed metagenomic sequencing on the layers of the old mat in 2010. All five samples were given unique multiplex identifier (MID) tags prior to pooling on the microtiter plate. Pyrosequencing was carried out as described above.

### SSU-rRNA processing

All analyses of the SSU-rRNA amplicons were conducted within the QIIME software package (Caporaso et al., 2010a). Raw 454 reads were quality filtered, dereplicated, and demultiplexed by removing Roche adapters, linkers, primers, and sample barcodes. Sequences shorter than 350 bp and longer than 550 bp were discarded. Next, to expedite chimera detection, we clustered identical reads using uclust (Edgar, 2010). One representative sequence of each cluster was aligned to the Greengenes core set with PyNAST 1.1 (DeSantis et al., 2006; Caporaso et al., 2010b). Chimeric sequences (7,804 total) were detected using the ChimeraSlayer algorithm and subsequently removed (Haas et al., 2011). To examine the effect of phylogenetic resolution on resulting diversity metrics, we used uclust to bin SSU-rRNA sequences into operational taxonomic units (OTUs) using a 97% similarity cutoff using a greedy algorithm. Singleton OTUs (those appearing in only one sample, or represented by only one sequence) were discarded. We assigned taxonomic identities to a sequence representing a particular OTU using the RDP naïve Bayesian classifier (Cole et al., 2009). These representative sequences were once again aligned to the Greengenes core set using PyNAST. Any sequences identified as belonging to archaea, eukarya, or chloroplasts were removed, as were singleton OTUs. The sequences were masked and used to build a phylogenetic tree with FastTree 2.1.4 using the GTR + CAT approximation for substitution rate heterogeneity (Stamatakis, 2006; Price et al., 2010). This software is optimized for phylogenetic inference from large datasets and makes use of nearest-neighbor interchange algorithms to significantly decrease computation time (Price et al., 2010).

### Metagenome processing

Shotgun sequences from the 2010 old mat layers were quality filtered and demultiplexed using QIIME’s split_libraries procedure. Sequences shorter than 75 bp were discarded. This filtering resulted in approximately 243.8 Mbp. These sequences were then submitted to MG-RAST for dereplication and screening against model organismal DNA (Meyer et al., 2008). Protein prediction was carried out in the MG-RAST pipeline using FragGeneScan (Rho et al., 2010). We used the categorization of the Kyoto Encyclopedia of Genes and Genomes (KEGG) for annotating our sequences (Kanehisa et al., 2012).

### Diversity estimation

Rather than using a univariate index to characterize the taxonomic richness and evenness of each mat layer, we computed the series of effective numbers, *^q^*D**^Z^(**p**), recently presented by Leinster and Cobbold (2012). This family of metrics is a simple extension of Hill numbers (Hill, 1973) that also account for the similarity between taxa, represented by a matrix **Z***_ij_*. These numbers are generalizations of particular diversity indices, and can be plotted against the parameter *q*, the sensitivity to rare taxa. At *q* = 0, the number equals the taxonomic richness *S*, if a naïve similarity matrix (the identity) is used, otherwise, it is a measure of phylogenetic diversity (Faith, 1992; Chao et al., 2010). At *q* = ∞, the naïve solution is the reciprocal of the Berger–Parker index and gives the inverse proportional abundance (*p*) of the most dominant taxa. To measure the phylogenetic diversity of a community, we used the equation.

$${}^{q}D^{z}\left(\pi \right)={\left(\underset{{}_{\pi_{{}_{\left(i,b\right)}>0}}^{i,b:i\in I_{b},}}{\sum }\pi_{\left(i,b\right)}{\left(Z\pi \right)}_{\left(i,b\right)}^{q-1}\right)}^{q∕\left(1-q\right)}$$

where π_(*i,b*)_ is the relative abundance of “historical species” (*i,b*), where *b* is a particular branch terminating in the subset of taxa present in the community: *i* ∈ {1, 2, 3,…, *S*}, *I_b_* ⊆ {1, 2, 3,…, *S*}, and ***Z****_(i,b)(j,c)_* is an asymmetric relatedness matrix (details in Leinster and Cobbold, 2012, Appendix). Additionally, we calculated ^0^*D***^Z^**(**p**) with ***Z*** = ***I***, which is simply the taxonomic richness of the community. For each layer, these values were calculated over a range of *q* (0–5) averaged over 100 subsamples rarefied to the smallest number of sequences in a layer (Old mat 2010, 10–15 mm, 1071 sequences).

Mean phylogenetic diversity (MPD) was estimated by calculating the average phylogenetic distance of all pairwise branch lengths. For examination of particular mono- and polyphyletic guilds within the overall phylogeny (e.g., Cyanobacteria, sulfur oxidizing bacteria), we trimmed the tree to only include particular clades and recalculated the distance matrix. These values were then averaged over 100 rarefactions normalized to the smallest sample.

### Null model analyses

We estimated the standard effect size of each community’s rarefied mean phylogenetic distance (MPD_SES_) by comparing it to the rarefied values of 999 randomized communities generated using null models. Because the statistical power to detect niche-based community assembly varies with the choice of null model, we assessed the agreement of three different randomization routines: (1) the independent swap algorithm, which shuffles the taxa/sample table; (2) the phylogeny.pool algorithm, which draws random samples from the phylogenetic distance matrix to populate the taxa/sample table; and (3) the taxa.labels algorithm, which shuffles the labels of a phylogenetic distance matrix (Kembel and Hubbell, 2006; Kembel, 2009). In null model three, we calculated phylogenetic diversity both by weighting the relative number of sequences obtained for each OTU as either 0 or 1 (presence/absence) or as *p_ij_*, the proportional relative abundance of the OTU in its respective sample. We acknowledge that measuring proportional abundance using sequence counts is not optimal, but it still provides a more realistic view of a community compared to presence/absence data. We assessed the robustness of our results by first comparing the results of null model three (taxa.labels) using three definitions of “taxa pools”: (1) the entire set of OTUs detected in all samples during all years, (2) the set of OTUs detected in each year, separately, and (3) the OTUs only found in each particular core. We did this with the expectation that phylogenetic dispersion is sensitive to the spatiotemporal scale of the taxa pool (Kembel and Hubbell, 2006; Swenson et al., 2006; Lessard et al., 2012). The first, and least conservative species pool assumes that all OTUs detected in the study are equally likely to colonize all mat layers, independent of year. The last, and most conservative pool assumes that the only OTUs capable of colonizing all three mat cores are those that were detected within all three cores to begin with. Additionally, because weak bifurcation support values in our phylogeny might bias our estimates of standard effect size, we tested the robustness of our findings by collapsing bifurcating nodes into polytomies if the nodes’ support values were below a certain threshold (50 and 80%). Samples were considered significantly overdispersed or clustered (α = 0.05) if they fell above or below 95% of the randomized communities’ values, respectively. We ran these tests for all OTUs detected in the sample, and for three functional guilds: (1) phylum Cyanobacteria (oxygenic phototrophs), (2) order Chromatiales (purple sulfur bacteria), and (3) orders Desulfobacterales, Desulfovibrionales, Syntrophobacteriales, and Clostridiales (anaerobic sulfate-reducers; Risatti et al., 1994).

Trait richness and dispersion of the OM-10 sample was measured using the function-level KEGG ortholog (KO) group, excluding hypothetical proteins at an *e*-value equal to or less than 10^−5^ with a minimum alignment length of 50 bp. To correct for sample size bias, we rarefied KO richness to the lowest number of KO genes detected in our sample (0–2 mm; 20,121). We followed the methods of Bryant et al. (2012) in re-sampling without replacement 1,000 simulated sets of KO genes for each community from the pool of KO genes detected throughout the mat core. We compared our observed KO richness to those of our simulated communities to assess whether or not layers contained more or fewer trait-encoding genes than would be expected by chance. We verified our findings by performing the same routine on non-supervised orthologous groups (NOGs), which are annotated algorithmically rather than by hand (Jensen et al., 2008), and clustered orthologous groups (COGs), which are manually curated (Tatusov et al., 1997). We also measured rarefied KO richness patterns for different groups of genes which may be ecologically important in structuring the microbial mat and compared these values to a randomized expectation. These gene categories included (1) total metabolism, (2) photosynthesis, (3) carbohydrate metabolism, (4) sulfur metabolism, (5) ABC transporters, and (6) oxidative phosphorylation. Significance was assessed using the rank test described for MPD_SES_.

### Statistical analyses

To statistically compare phylogenetic richness between communities, we calculated the unweighted SSU-rRNA UniFrac distances between samples, rarefied to the sample with the lowest number of sequences, and clustered using Ward’s minimum variance method (Lozupone and Knight, 2005). Statistical significance was assigned to clusters as determined by approximately unbiased (AU) *p*-values calculated from 10,000 multiscale bootstrap dendrograms (Shimodaira, 2004). We used the permutation-based analysis of variance ADONIS to test the null hypotheses of whether differences existed in community OTU composition between years, sites, and depth (Anderson, 2001). We used linear regression to test for a relationship between our Hill diversity measures and pH, depth, chl *a*, Bchl *a*, sulfide, and oxygen concentrations. Because our data appeared non-linear in exploratory analyses, we compared linear and power law (log–log-transformed) fits for the same data and selected the top model based on its coefficient of determination. We calculated regressions both with and without covariates for the age of the mat sample and the year in which it was collected. All quantitative analyses were carried out in R 2.14 (R Development Core Team, 2011) using the packages “vegan 2.0” (Oksanen et al., 2012), “picante 1.3” (Kembel et al., 2010), and custom scripts for rarefaction and diversity profiling (Bryant^1^; Cobbold^2^; Armitage^3^).

## Results

**Figure 2:**
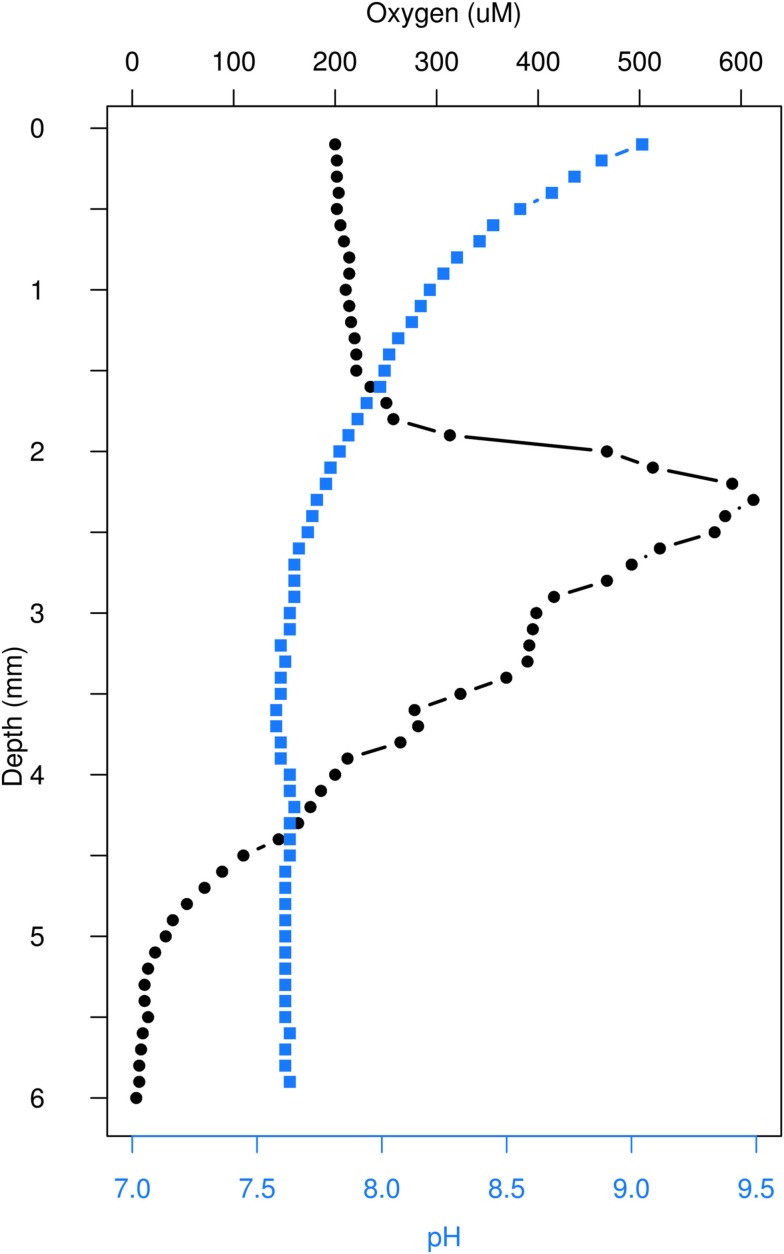
***In situ* microsensor depth profile for oxygen concentration and pH**.

Our measurements of variation in abiotic conditions with depth demonstrate clear gradients. Our oxygen profile showed the predicted spike in concentration between 2 and 3 mm, and decreased to trace levels past 5 mm (Figure 2). Likewise, pH declined with depth to 3 mm and remained constant to 6 mm (Figure 2).

**Figure 3:**
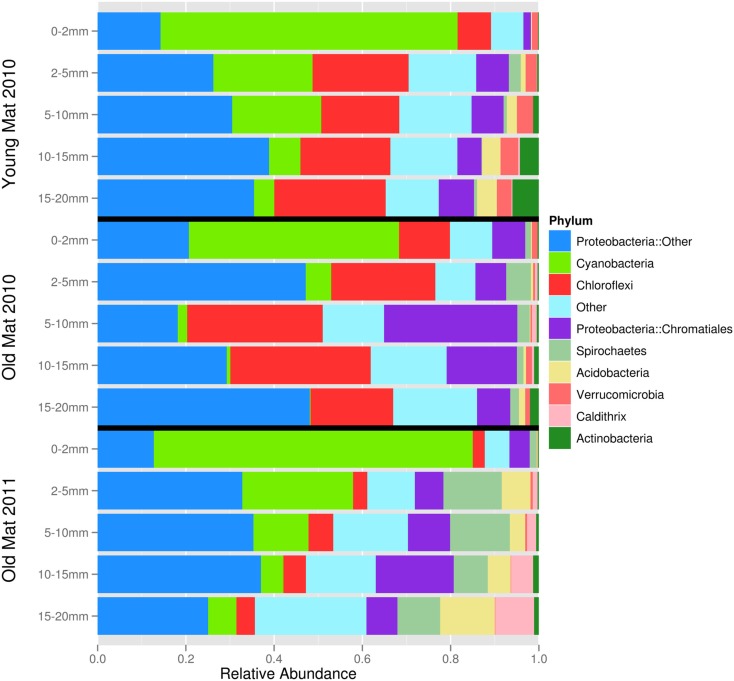
**Phylum-level community composition for each mat layer and core sample**. ‘Proteobacteria::Other’ includes orders Syntrophobacterales, Rhodospirillales, Rhodobacterales, Desulfobacterales, Campylobacterales, Oceanospirillales, Myxococcales, Desulfovibrionales, Salinisphaerales, and Rhizobiales. Phyla in “Other” category (<1% relative abundance) include Bacteroidetes, Chlamydiae, Chlorobi, Firmicutes, Fusobacteria, Gemmatimonadetes, Lentosphaerae, Nitrospirae, Planctomycetes, Tenericutes, Thermi, and candidate divisions ABY1, BRC1, GN02, GN04, GN06, GN12, HYD24-12, KSB1, LCP89, MSBL6, MVP-15, NKB19, OP8, OP9, OP11, SAR406, SC4, TG3, TM6, TM7, WPS-2, WS1, WS3, ZB2.

**Figure 4:**
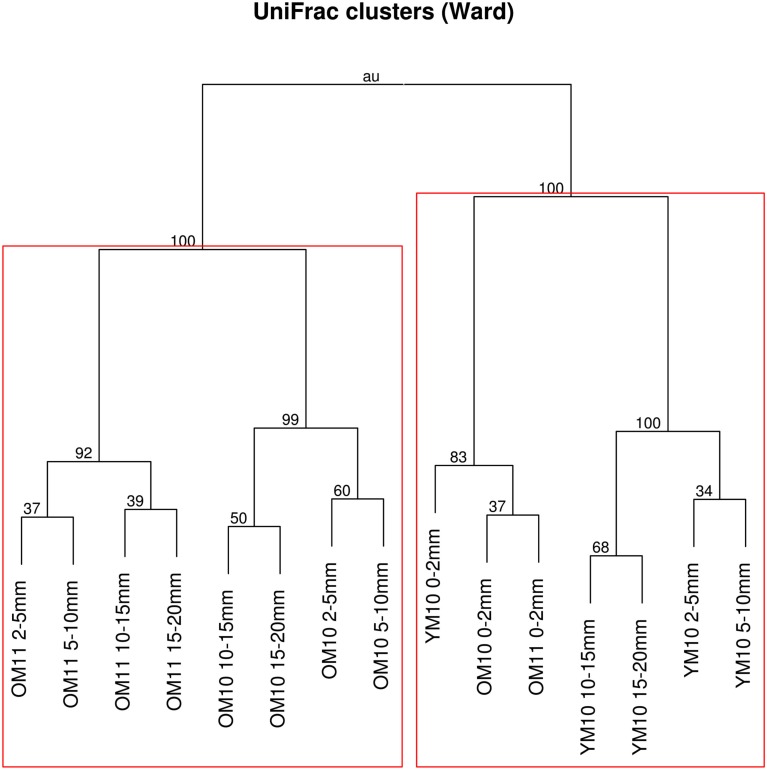
**Dendrogram of UniFrac distances clustered using Ward’s minimum variance method**. Node labels are AU *p*-values. Values greater than 95 indicate significant clusters. Rectangles indicate the deepest significant clusters within each clade. OM, old mat; YM, young mat; 10, 2010; 11, 2011.

After quality filtering, dereplication, and singleton removal, we were left with a total of 91,392 SSU-rRNA sequences representing 3,503 OTUs clustered at 97% sequence similarity. The top layers of each cluster were dominated by Cyanobacteria of the family Oscillatoriales, while the lower clusters were heavily dominated by Proteobacteria, including many OTUs classified to the family Chromatiales (Figure 3). UniFrac clustering revealed that samples were clustered by year and age (Figure 4). Two statistically significant clusters were identified: one containing all but the top layers of the old mats, and one containing the top layers of all mats and the young mat (AU test; *p* < 0.001; Figure 4). We rejected the null hypotheses that communities were compositionally identical (α = 0.05) across years (*F*_1,13_ = 4.20, *p* < 0.005), ages (*F*_1,13_ = 3.34, *p* < 0.005), and depth (*F*_1,13_ = 2.72, *p* < 0.005). However, these three effects only explained 50.6% of the variance in our data.

**Figure 5:**
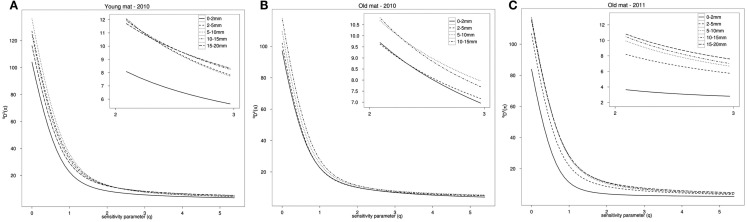
**Rarefied phylogenetic diversity profile, *^q^D*^Z^(π), for (A) the young mat sample, (B) the old mat 2010 sample, and (C) the old mat 2011 sample**. Increasing parameter *q* decreases the metric’s sensitivity to rare taxa and becomes a measure of evenness. Inset box shows values 2 ≤ *q* ≤ 3.

Taxonomic richness, ^0^*D*(**p**), and phylogenetic richness, ^0^*D***^Z^**(**π**), were both positively associated with depth on the log–log scale, and this result was significant (*p* < 0.001, *R*^2^ = 0.64; *p* < 0.0001, *R*^2^ = 0.75). Profiles of *^q^*D*^**Z**^*(π) show that the top layer of each mat had the lowest phylogenetic richness (*q* = 0) and evenness (*q* > 0). Overall phylogenetic richness was greatest in the young mat, with the exception of the top layer, which had the lowest phylogenetic and taxonomic richness of the three cores (Figure 5).

**Figure 6:**
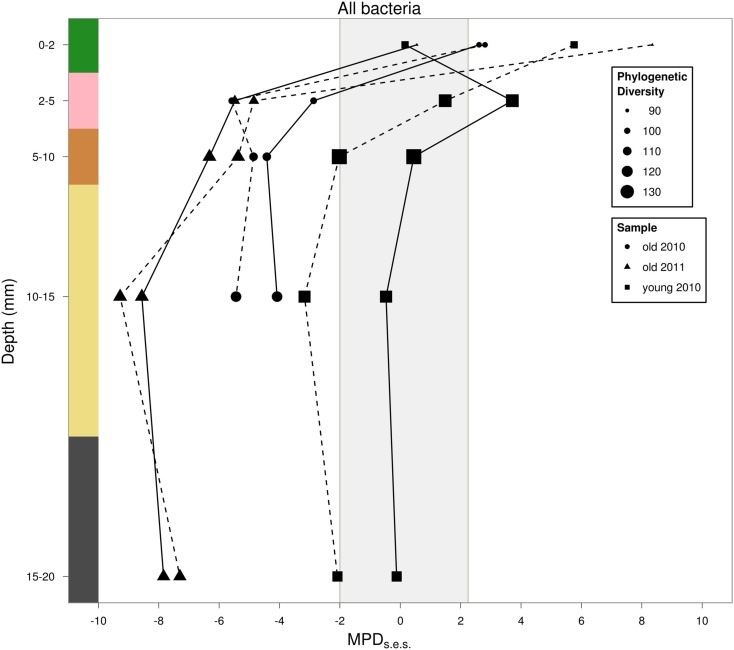
**Estimated rarefied phylogenetic dispersion with depth**. Solid lines are results using taxa pool 1 (all years, all cores) as the regional pool, dashed lines are results using taxa pool 3 (individual cores) as the regional pool. Point size is scaled to phylogenetic diversity, ^0^*D***^Z^**(π). Values falling below the shaded region indicate statistically significant phylogenetic clustering. Values above this region indicate phylogenetic overdispersion.

Phylogenetic clustering increased linearly with depth when mat age was included as a covariate (*p* < 0.05, *R*^2^ = 0.65; Figure 6). The old mat samples from 2010 to 2011 showed similar patterns in phylogenetic dispersion, with positive MPD_SES_ values in the top layer and significantly clustered communities in the underlying layers. Additionally, we found evidence for an increase in phylogenetic clustering with mat age for depths >2 mm (Figure 6). The young mat was similarly patterned, but the significance of the clustering was less than that of the older mats. Our results did not change when phylogenetic bifurcations were collapsed into polytomies at 50 and 80% support values. Our choice of null model did not change most of our results, but the independent swap algorithm dampened the strength of statistical significance compared to the phylogeny-shuffling approaches (Table A1 in Appendix). Compared to our first taxa pool (all OTUs in all years and samples), pools 2 (within-year), and 3 (within-core) altered magnitude of statistical significance for many of the samples (Table A1 in Appendix). There were no differences in outcomes between relative abundance-weighted and censored randomizations (Table A1 in Appendix). Chromatiales (purple sulfur bacteria) exhibited no significant phylogenetic dispersion in any layer. The same was true for Cyanobacteria, except for one significantly clustered community in the top layer of the young mat (MPD_SES_ = −2.11, rank = 15, *p* < 0.05). Sulfate reducing bacteria were phylogenetically clustered in the young mat, but only significantly so for the 2–5 mm (pink) layer (MPD_SES_ = −3.79, rank = 7, *p* < 0.01).

**Figure 7:**
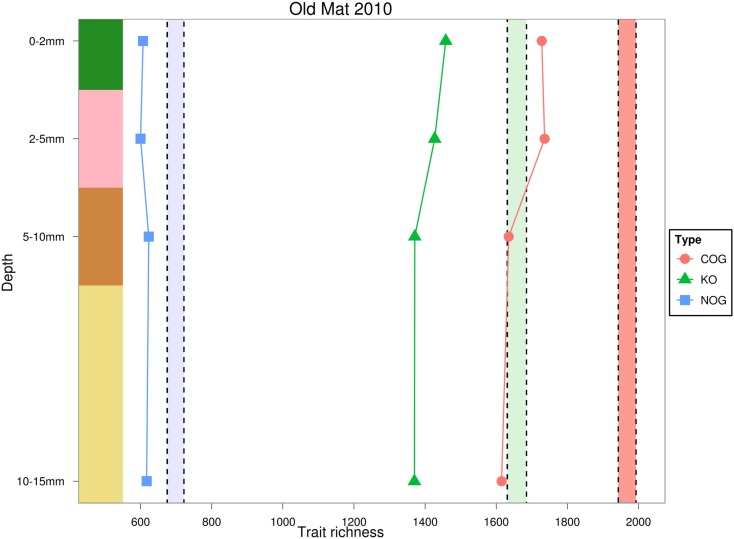
**Estimated rarefied trait richness in the OM-10 core using three different orthologous gene annotations**. Values falling below or above their similarly colored shaded regions indicate significantly fewer (α = 0.05) or greater trait richness than expected under 1,000 null model randomizations, respectively.

**Figure 8:**
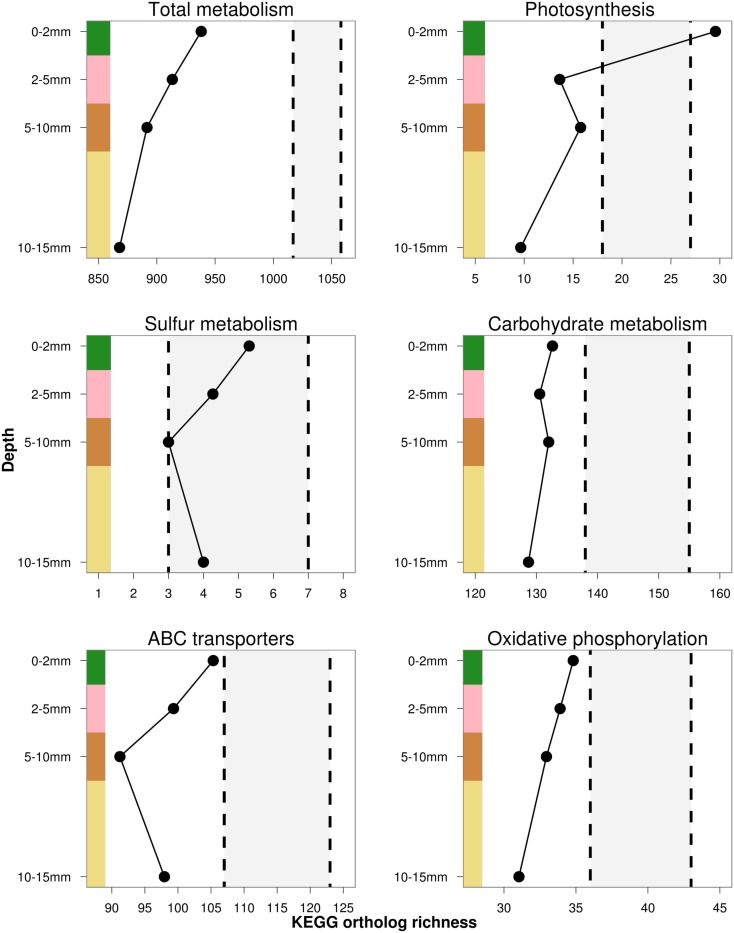
**Estimated rarefied KEGG trait richness in the OM-10 core for a subset of functional gene categories**. Values falling below or above the shaded regions indicate significantly fewer (α = 0.05) or greater trait richness than expected under 1,000 null model randomizations, respectively.

Trait richness, measured by rarefied KO and COG annotations, decreased with depth, and appeared to negatively covary with taxonomic and phylogenetic richness. Using the NOG gene annotation, functional richness appeared to slightly increase in the third and fourth layers, but could not be statistically evaluated with only four data points. Total trait richness was significantly lower than expected under a randomized sample for all layers and all ortholog databases, indicating fewer traits were present in each layer than expected by chance (Figure 7). Photosynthesis-related KO genes decreased with depth, and were significantly greater than expected by chance in the top layer, and reduced in the underlying layers (Figure 8). Richness of metabolic KO genes decreased with depth. Genes related to sulfur metabolism and carbohydrate metabolism remained relatively constant with depth, though the former category had very few KO hits. Genes encoding ABC transporters decreased in richness to the third (brown) layer, and then increased in the bottom layer. Genes coding for oxidative phosphorylation steadily declined with depth.

## Discussion

### Community composition

Using a combination of bacterial SSU-rRNA amplicons and metagenomics, we quantified patterns in taxonomic, phylogenetic, and trait diversity within a salt marsh microbial mat. Our results, taken in concert with other studies, suggest cyanobacterial mats contain a very diverse community with large variation at the millimeter-scale (Ley et al., 2006; Villanueva et al., 2007; Kunin et al., 2008; Dillon et al., 2009). This variation appeared to be primarily driven by light limitation with depth. At the surface, light drives oxygenic photosynthesis in organisms possessing chlorophyll *a* and favors the oxidation of water to oxygen. As light becomes limited at depths greater than 2 mm, phototrophs possessing bacteriochlorophylls dominate, such as the purple bacteria (e.g., orders Rhodospirillales and Chromatiales) and the green sulfur bacteria (order Chlorobiales). These organisms use sulfide, hydrogen, and organics as reducing agents, scavenging it from the anaerobic reduction of sulfate carried out in the underlying layers. Our finding that all of the young mat’s layers most closely resembled the top green layers of the developed mats is not surprising, considering that in the absence of thick EPS layers, light probably penetrates much deeper into the mat. While the young mat sample did not display laminae, it still contained numerous taxa identified as sulfate-reducers, as well as potential sulfur oxidizing bacteria. This result is in agreement with other studies of intertidal mats, which demonstrate patterns of succession initiated by *Oscillatoria* spp. and other oxygenic phototrophs (Stal et al., 1985; Bolhuis and Stal, 2011). These early colonizers contribute to the formation of the underlying layers by stabilizing the sediment and making available organic carbon to the heterotroph communities, whose activities in turn create anoxic conditions and permit dissimilatory sulfate reduction and photosynthetic oxidation of hydrogen sulfide as life history strategies (Herbert and Welsh, 1994).

### Diversity patterns

In all three mat cores, phylogenetic and taxonomic richness was lowest in the top layer. Similar patterns have been found in marine systems (Stevens and Ulloa, 2008; Kembel et al., 2011; but see Bryant et al., 2012), hypersaline lakes (Humayoun et al., 2003), subtidal sand (Böer et al., 2009), and hypersaline microbial mats (Dillon et al., 2009). Because the sharpest increase in diversity occurred during the transition between the first and second layers, we hypothesize that the top layer of the mat may be a habitat unfavorable to the majority of OTUs detected in our samples due to a combination of UV irradiation, temperature, and flood scouring (Calkins and Thordardottir, 1980; Garcia-Pichel and Castenholz, 1994; Ibelings and de Winder, 1994; Bossio and Scow, 1998). Because these factors fluctuate on a daily cycle, and because these communities undergo massive diel migrations, the daytime top layer of the mat is likely composed of stress-tolerant aerobes, while at night, microaerophiles and UV-sensitive taxa temporarily migrate to the top layer (Villanueva et al., 2007; Dillon et al., 2009). Niche theory predicts that areas of higher resource heterogeneity will support a greater diversity of taxa (MacArthur and Levins, 1967; but see Stevens and Carson, 2002). Thus, if a particular mat layer has a greater diversity of resources, we might expect an equally proportional diversity of genes relating to resource use. However, we did not find evidence of a decreasing diversity with depth of metabolic strategies measured by overall metabolic KO genes, nor genes encoding for carbohydrate metabolism, and ABC transporters, suggesting that an increase in resource heterogeneity does not explain the correlation between diversity and depth. Competition theory presents an alternative mechanism to explain the increase in diversity with mat depth. We speculate that the pool of nutrients available for assimilation (primarily *N* and *P*) may be limiting to heterotrophs in the oxic layer due to the large biomass of primary producers such as heterocystous Cyanobacteria and diatoms (Herbert and Welsh, 1994; Camacho and de Wit, 2003; Jonkers et al., 2003; Yannarell and Paerl, 2007). If competition for limiting nutrients, rather than competition for sources of C and energy, are resulting in competitive exclusion then we might expect competitive exclusion should not be as dominant a force deeper in the mat, allowing more taxa to coexist (Hutchinson, 1961). Indeed, we found indirect support for competition being greater in the top mat layer than in underlying layers based on phylogenetic dispersion measurements. At the domain level, the top layers of each core often showed phylogenetic overdispersion, meaning OTUs detected in this layer were less related than would be expected by chance. These results match those of Bryant et al. (2012), who found significant phylogenetic overdispersion only in the uppermost photic layers of their pelagic depth series, a region also dominated by primary production and aerobic metabolism. In soils, Horner-Devine and Bohannan (2006) found a negative relationship between total organic carbon and the phylogenetic relatedness of the community. If the assumption of phylogenetic niche conservatism holds for the domain bacteria, and if competitive interactions are greatest among sister clades, then phylogenetic overdispersion can be taken as evidence for limiting similarity within a set of interacting taxa (MacArthur and Levins, 1967; Webb et al., 2002). Currently, there is limited evidence for either assumption in microbial communities, and this remains a fertile area for research (Boyd et al., 2010; Mayfield and Levine, 2010; Violle et al., 2011).

The qualitatively negative relationship between taxonomic/phylogenetic richness and trait richness measured via KO and COG pathways was unexpected. The KEGG and COG databases we used to annotate gene function in our samples were obtained from sequenced genomes, which are heavily biased toward model organisms and those from well-studied environments. Thus, the decrease in trait richness we observe may be due to annotation bias if deeper mat layers contain more organisms with poorly characterized genes and pathways. Our data do not support this claim, however, as we did not observe either a decrease in the proportion of unassigned reads nor an increased mean *e*-value with depth. Differences in OTU abundance between layers might also impact the observed pattern of trait richness. If the layer with the highest taxonomic richness is also the least even of the samples, the set of genes identified in that community will be biased toward the dominant taxa. Our diversity profiles clearly show that both diversity and evenness increase with depth, and so trait bias toward dominant OTUs cannot explain the decrease in trait richness that occurs with mat depth. Finally, our omission of archaeal reads from the amplicon library may explain the pattern, since Archaea are probably proportionally more abundant in the anaerobic layers. However, they do not exhibit as wide a physiological breadth as the domain Bacteria and are not expected to strongly bias measurements of trait diversity.

That our data show clear patterns of phylogenetic clustering with depth suggests that niche-based habitat filtering impacts community assembly as oxygen and light become limited. This claim is strengthened by our finding of significantly fewer KO genes in each layer than are expected by chance. When particular functional guilds of taxa were examined, however, this pattern dissolved, indicating that this clustering may only be apparent at very broad taxonomic levels, or in clades which we did not investigate. Other studies have also found an effect of taxonomic resolution on community clustering patterns (Horner-Devine and Bohannan, 2006; Pontarp et al., 2012). Habitat filtering in this community may be driven by the phylogenetic conservatism of phenotypes capable of anaerobic respiration. However, the metagenomic approach does not allow us to test this prediction directly due to both our inability to match taxon and gene, and our inability to match genotype with phenotype. Nonetheless, phylogenetic clustering patterns dominate the literature, and appear to covary with a number of factors, such as chlorophyll *a* content, organic carbon, nitrate (Horner-Devine and Bohannan, 2006), ocean depth (Kembel et al., 2011; Bryant et al., 2012), elevation (Bryant et al., 2008; Wang et al., 2012), and salinity (Barberán and Casamayor, 2010). More generally, the principle of habitat filtering has been the basis for enrichment culturing of microbes since the early days of microbiology, and it is not surprising that different habitats, characterized by different metabolic substrates and abiotic conditions, will selectively filter out all but a subset of taxa in the same way as a defined medium in a culture flask. One model of phylogenetic succession predicts that if the traits of early colonizers are phylogenetically conserved, then habitat filtering should give way to facilitative and competitive interactions as succession progresses, which should manifest as a switch from phylogenetically clustered to random/overdispersed communities with time (Verdú et al., 2009). In our samples, the increased phylogenetic clustering with mat age at depths below 2 mm, along with the compositional clustering among the young mat and the top layers of the old mats, suggests the increased importance of habitat filtering with succession, as poorly adapted initial colonizers are lost from underlying layers while taxa more suited to the changing abiotic conditions are recruited into the habitat.

### Null model considerations

In many cases, our choice of null model affected the interpretation of our analyses. By decreasing the size of our regional taxa pools from the entire suite of OTUs in a sample to the set of OTUs in one particular mat layer, we were limiting the dispersal potential for taxa not shared among samples. Little is known about the scale at which microbes disperse (Whitaker et al., 2003), but we found relatively similar results between the two most extreme taxa pools (Table A1 in Appendix). It is likely that no two taxa have the same likelihood of occupying a habitat, and future studies should consider a spatially explicit metacommunity approach to identifying more realistic taxa pools (Lessard et al., 2012; Peres-Neto et al., 2012). Likewise, we found that the independent swap algorithm decreased the effect sizes of MPD. This null model has been identified as being the most conservative in detecting niche-based community assembly in simulations (Kembel, 2009). This randomization routine, compared with the two phylogeny-based approaches, did not identify overdispersion in our 0–2 mm layers. The statistical power to detect limiting similarity in communities requires testing, as do alternative null models which incorporate the effects of real ecological processes (Gotelli and Ulrich, 2012). By weighting our randomizations by a taxon’s relative abundance, we were assuming that the probability of colonization in a mat layer was proportional to the taxon’s abundance in the community, while presence/absence data give all taxa an equal probability (Kembel and Hubbell, 2006). In reality, the true dispersal probability for a taxon lies somewhere in between these two extremes, but has yet to be quantified so that an appropriate null model can be developed. In the absence of more sophisticated, semi-mechanistic null models, we advocate a pluralistic view of null modeling schemes focusing efforts on interpretation of a suite of models in light of their constraints and assumptions. Additionally, most, if not all, of the assumptions surrounding null models and community assembly (e.g., dispersal scale, niche conservatism) have yet to be assessed for the majority of organisms, microbial or otherwise, and we strongly urge ecologists to interpret their results in the light of these knowledge gaps.

## Conclusion

Microbial mats exhibit marked variation in diversity at scales much smaller than typically considered in ecological studies. Our observations of phylogenetic and trait clustering within the layers of a salt marsh microbial mat indicate habitat filtering as the main driver for community assembly. However, the effects of biotic interactions such as competition and syntrophy cannot be dismissed as alternative factors affecting community structure, especially in the mat’s topmost Cyanobacteria-dominated layer. Nevertheless, until rigorous manipulative studies are carried out to test the assumptions of phylogenetic niche conservatism and dispersal limitation in bacteria, results from community phylogenetic approaches must be interpreted with caution. We suggest that these types of studies be carried out using microbial mat communities, which have historically proven tractable to both laboratory and field experimentation.

## Conflict of Interest Statement

The authors declare that the research was conducted in the absence of any commercial or financial relationships that could be construed as a potential conflict of interest.

## Footnotes

^1^http://openwetware.org/wiki/User:Jessica_A._Bryant

^2^http://www.maths.gla.ac.uk/∼cc/supplements/diversity.html

^3^http://github.com/darmitage

## Appendix

**Table A1:** **Rarefied MPD_SES_ values for all bacteria, Cyanobacteria, purple sulfur bacteria, and sulfate reducing bacteria**.

Sample	Group	Taxa.labels			Ind. Swap	Phylo. Pool	RelAbund
		Depth	Pool 1	Pool 2	Pool 3	Pool 1	Pool 1	Pool 1
YM-2010	All bacteria	0–2	0.16	5.76*	−0.55	−0.68	0.1	−0.02
		2–5	3.71*	1.48	1.82	2.03	3.56*	3.63*
		5–10	0.45	−2.02*	−1.54	0.54	0.4	0.43
		10–15	−0.47	−3.17*	−1.3	−0.03	−0.49	−0.52
		15–20	−0.12	−2.08*	−3.4*	0.13	−0.16	−0.14
	Cyanobacteria	0–2	−2.11*	−0.01	−2.07		
		2–5	−1	−0.45	−0.63		
		5–10	−0.312	−0.64	0.24		
		10–15	−0.86	0.79	−0.53		
		15–20	−0.429	0.89	0.21		
	Purple sulfur bacteria	0–2	−1.66	−0.82	−1.63		
		2–5	−1.13	−0.17	−0.4		
		5–10	0.18	−0.32	1.1			
		10–15	−0.96	−1.22	−0.53		
		15–20	−1.26	−1.11	−1.22		
	Sulfate reducing bacteria	0–2	NA	NA	NA		
		2–5	−2.71	−1.25	−0.32		
		5–10	−3.78	−2.53	−1.6		
		10–15	−1.88	−0.54	0.27		
		15–20	−2.5	−0.16	−0.29		
OM-2010	All bacteria	0–2	2.61*	2.81*	3.99*	1.41	2.6*	2.61*
		2–5	−2.87*	−5.58*	−2.17	−1.41	−2.72*	−2.77*
		5–10	−4.42*	−4.86*	−4.12*	−2.29*	−4.31*	−4.39*
		10–15	−4.08*	−5.44*	−3.73*	−2.13*	−4.09*	−4.1*
		15–20	NA	NA	NA	NA	NA	NA
	Cyanobacteria	0–2	−1.18	−0.14	−2.13		
		2–5	0.74	−1.18	0.54		
		5–10	0.80	0.33	0.46		
		10–15	NA	NA	NA		
		15–20	NA	NA	NA		
	Purple sulfur bacteria	0–2	−0.09	1.83	−0.75		
		2–5	−0.95	2.1	0.19		
		5–10	−0.75	1.97	−0.99		
		10–15	−0.20	1.13	−0.19		
		15–20	−0.19	1.02	0.12		
	Sulfate reducing bacteria	0–2	NA	NA	NA		
		2–5	−1.66	−0.76	0.04		
		5–10	0.29	0.54	0.32		
		10–15	0.69	0.82	0.94		
		15–20	NA	NA	NA			
OM-2011	All bacteria	0–2	0.55	8.34*	6.49*	0.33	0.56	0.52
		2–5	−5.48*	−4.86*	−1.69	−2.61*	−5.62*	−5.5*
		5–10	−6.32*	−5.36*	−2.03	−2.98*	−6.46*	−6.35*
		10–15	−8.56*	−9.28*	−7.24*	−4.04*	−8.65*	−8.61*
		15–20	−7.84	−7.29*	−5.14*	−3.84*	−7.92*	−7.92*
	Cyanobacteria	0–2	−1.211	1.22	−1.5		
		2–5	−1.06	−0.99	−0.96		
		5–10	−0.156	−0.49	−0.65		
		10–15	−0.974	−1.23	−0.79		
		15–20	−1.266	1.03	−0.46			
	Purple sulfur bacteria	0–2	−0.48	0.54	0.02		
		2–5	−0.39	−0.69	−1.09		
		5–10	−0.16	−0.16	−0.6		
		10–15	−0.02	0.91	−0.1		
		15–20	−0.19	−0.95	−1.45			
	Sulfate reducing bacteria	0–2	−0.08	−0.51	−1.02		
		2–5	0.54	−0.24	0.12		
		5–10	0.70	0.61	0.56		
		10–15	1.11	−0.07	1.22		
		15–20	0.94	0.27	0.97		

*Values are presented for three species pools, and four null models, including abundance-weighted randomizations (see text for details). *Indicates significant (*p* < 0.05) phylogenetic clustering (negative values) or overdispersion (positive values) based on rank test*.
